# Reviewed article: Lerm BR, Pletsch V, Géa MFF, Santos IS, Regner AP, Giugliani C. Social vulnerability of children and adolescents who are victims of sexual violence assisted in a specialized service: descriptive study, Porto Alegre and metropolitan region, 2018. Epidemiol Serv Saude. 2026:35;e20240780

**DOI:** 10.1590/S2237-96222026v35e20240780.b

**Published:** 2026-10-30

**Authors:** Osmar Cardoso

**Affiliations:** 1 Universidade Federal do Piauí, Teresina, PI, Brazil Universidade Federal do Piauí Teresina PI Brazil

## Peer review B

## Questionnaire

Does the manuscript contain new and significant information to justify publication? Yes

Does the Abstract (Summary) clearly and accurately describe the content of the article? No

Is the problem significant and concisely stated? Yes

Are the methods described comprehensively? Yes

Are the interpretations and conclusions justified by the results? No

Is adequate reference made to other work in the field? Yes

## Manuscript Structure:

Length of article is: Adequate

Number of tables is: Adequate

Number of figures is: Adequate

Please state any conflict(s) of interest that you have in relation to the review of this paper: None

Interest: Good

Quality: Average

Originality: Good

Overall: Good

Recommendation: Major Revision

## Comments

Minhas preocupações com o artigo são:

1) É preciso uma revisão criteriosa da forma da escrita e apresentação dos dados (ex: pág.5, linha 48, quando fala da violência contra as meninas não se apresenta o número, e se fala o óbvio em seguida que das meninas foi mais intrafamiliar, e em seguida dos meninos que foi mais extrafamiliar). É necessário uma revisão da forma da escrita.2) Apesar do estudo não apresentar a relação de maior violência com maior IVS ou UDH, os autores utilizam referências que corroboram isso, embora o estudo apresentado mostre outras informações e os autores não justificam essa discrepância (que pode ser verdade ou não) nem sugerindo causas que justifiquem esses achados locais.

3) Na figura apresentada, ele mostra Porto Alegre em separado da Região Metropolitana o que tira o parâmetro visual da comparação, além disso, não identifica os municípios na figura e nem os bairros de Porto Alegre.

4) Na figura, é evidenciado mais uma vez que a violência não está relacionada ao UDH e na discussão, esses fatos parecem ignorados.

